# Supplementary material for: Content-rich biological network constructed by mining PubMed abstracts
Source: BMC Bioinformatics. 2004 Oct 8;5:147. doi: 10.1186/1471-2105-5-147 (PMC528731; doi:10.1186/1471-2105-5-147)
Supplement: Additional File 2 — The original results of the above study (non-essential files are deleted to keep the file size under the limit set by BMC bioinformatics). [file 1471-2105-5-147-S2.bz2 › chilibotAdditionalFile2/dip20/304ID9325095E1301/html/APP_ACHE.html]

 


 **APP** and **ACHE** 
  
Found 98 abstracts in PubMed, retrieved 20.  
 

 What does Google say? 
 PDF only 
| .edu only 

---

**Interactive relationship** (e.g. stimulation, inhibition, etc)

**Stimulatory relationship**- Amyloid beta  [ **APP** ]  increases acetylcholinesterase  [ **ACHE** ]  expression in neuroblastoma cells by reducing enzyme degradation.  Ref: 12871588 J Neurochem, 2003
- Involvement of oxidative stress in the enhancement of acetylcholinesterase  [ **ACHE** ]  activity induced by amyloid beta  [ **APP** ]  peptide.  Ref: 12507730 Neurosci Res, 2003
- In particular, it appears that  **AChE**  may directly interact with amyloid beta  [ **APP** ]  in a manner that increases the deposition of this peptide into insoluble plaques.  Ref: 12669110 Drugs Today Barc , 2003
**Neutral relationship**- Amyloid fibril formation by a synthetic peptide from a region of human acetylcholinesterase  [ **ACHE** ]  that is homologous to the Alzheimer s amyloid beta  [ **APP** ]  peptide.  Ref: 12427014 Biochemistry, 2002
- A region near the C terminus of human acetylcholinesterase  [ **ACHE** ]   **AChE**  is weakly homologous with the N terminus of the Alzheimer s disease amyloid beta  [ **APP** ]  peptide.  Ref: 12427014 Biochemistry, 2002
- Alterations in  **APP**  metabolism have recently been proposed to play a key role in the long lasting effects of  **AChE**  inhibitors.  Ref: 12769797 Curr Drug Target CNS Neurol Disord, 2003

**Non-interactive relationship** (e.g. studied together, co-existance, homology, etc.)

- Altered glycosylation of acetylcholinesterase  [ **ACHE** ]  in  **APP**  SW Tg2576 transgenic mice occurs prior to amyloid plaque deposition.  Ref: 12065653 J Neurochem, 2002
- Further work is warranted to elucidate the exact roles in  **APP**  metabolism of the  **AChE**  inhibitors used in AD therapy at present.  Ref: 12769797 Curr Drug Target CNS Neurol Disord, 2003
- In this study we report that amyloid beta  [ **APP** ]  peptides Abeta increased  **AChE**  levels in both neuronal and astrocytic primary cultures, supporting the possibility that both neurons and glia may make a direct contribution to the pool of  **AChE**  seen around amyloid deposits in the AD brain.  Ref: 12591148 Brain Res, 2003
- Glyc  **AChE**  is increased in  **APP**  SW Tg2576 transgenic mice prior to amyloid plaque deposition, which suggests that Glyc  **AChE**  may be an early marker of AD.  Ref: 12704813 J Neurosci Res, 2003
- This review surveys recent data from in vivo and in vitro studies that have contributed to our understanding of the role of  **AChE**  inhibitors in  **APP**  processing.  Ref: 12769797 Curr Drug Target CNS Neurol Disord, 2003
- Based on the data of this study, we concluded that the number of intrinsic cardiac neurons may be approximated  [ **APP** ]  in the total heart preparation via counting and measuring of intramural ganglia, contours of which are well discernible following a histochemical reaction for  **AChE** .  Ref: 11936191 Ann Anat, 2002
- The nicotinic nAChRs and muscarinic mAChRs acetylcholine receptors and acetylcholinesterase  [ **ACHE** ]   **AChE**  activity were studied in the brains of  **APP**  SWE transgenic mice Tg and age matched nontransgenic controls Tg that were between 4 and 19 months of age.  Ref: 12093166 Mol Cell Neurosci, 2002
